# Effectiveness and safety of tenosynovitis of the long head of the biceps brachii with acupuncture: a protocol for a systematic review and meta-analysis

**DOI:** 10.1186/s13063-020-04800-6

**Published:** 2020-10-20

**Authors:** Rongrong Li, Yongliang Jiang, Renjie Hu, Xiaofen He, Jianqiao Fang

**Affiliations:** Key Laboratory of Acupuncture and Neurology of Zhejiang Province, The Third Clinical Medical College of Zhejiang Chinese Medical University, Hangzhou City, 310053 Zhejiang Province China

**Keywords:** Acupuncture, Tenosynovitis, LHB, Brachii, Meta-analysis

## Abstract

**Background:**

Tenosynovitis of the long head of the biceps (LHB) brachii is a common disease in patients over 40 years old. It can always result in chronic anterior shoulder pain and limited function. Acupuncture is one of most popular conservative treatment methods, and increasing studies indicate that it has remarkable therapeutic effects on the tenosynovitis of LHB brachii. However, the effectiveness and safety of acupuncture for treating tenosynovitis of LHB brachii remain largely uncertain. In our study, we will perform the first systematic review and meta-analysis to explore the effectiveness and safety of acupuncture on the tenosynovitis of LHB brachii.

**Methods:**

We will search the randomized controlled trial (RCT) literatures involving acupuncture for treating tenosynovitis of LHB brachii in eight electric databases, including PubMed, Web of Science, EMBASE, the Cochrane Library, Chinese National Knowledge Infrastructure (CNKI), Chinese Biomedical Literature Database (CBM), Wanfang Database, and Technology Periodical Database (VIP). We will define the visual analog scale (VAS), the Melle score of shoulder joint functional activity, and the ability assessment of daily living activities (ADL) as the primary outcomes. Besides quality of life, adverse events caused by acupuncture will be regarded as the secondary outcomes. Quality assessment of the included studies will be independently performed according to the Cochrane Risk of Bias tool. Meanwhile, the level of evidence for results will be assessed by using the Grading of Recommendations Assessment, Development, and Evaluation (GRADE) method. All analyses will be conducted by using the RevMan software V5.3.

**Results:**

From the study, we will ascertain the effectiveness and safety of acupuncture treatment on tenosynovitis of LHB brachii.

**Conclusion:**

The conclusion of this study will confirm the effectiveness and safety of acupuncture in the treatment of tenosynovitis of LHB brachii, which can provide new evidence to guide appropriate interventions on tenosynovitis of LHB brachii with acupuncture in the future.

**Ethics and dissemination:**

Ethical approval is not required because no individual patient data are collected. This review will be published in a peer-reviewed journal and presented at an international academic conference for dissemination.

**Trial registration:**

PROSPERO registration number CRD42020167434. Registered on April 28, 2020.

## Introduction

Tenosynovitis of LHB brachii is an inflammatory tendinitis that occurs as the tendon courses along its constrained path within the bicipital groove of the humerus. It may be caused by overuse from athletics requiring the overhead motion or degeneration from the normal aging process [1, 2]. However, tendinopathy of the LHB brachii encompasses a broad spectrum of pathology ranging from mild tendinopathy to rupture, which includes the inflammatory, degenerative, overuse-related, and traumatic causes [3, 4]. Similar to other types of biceps tendinopathy, patients with tenosynovitis of LHB brachii have a deep, throbbing ache in the anterior shoulder. Meanwhile, repeated overhead motion of arm may exacerbate the symptoms of intolerant pain, which can lead to movement restriction [5]. In terms of epidemiology, this disease is commonly found in adults over 40 years old, and it always has the symptoms of chronic anterior shoulder pain and limited function. Therefore, tenosynovitis of LHB brachii severely affects the quality of patients’ life, mainly manifested as the emotional disturbances, the pain localized in the bicipital groove and usually aggravated at night, and the movement of shoulder joint disorders caused by local adhesion.

The main purpose of tenosynovitis of LHB brachii treatment is to control the process of inflammatory response, relieve pain, and restore the range of motion of the whole shoulder joint. Currently, there are two main methods to treat tenosynovitis of LHB brachii including conventional treatments and surgical interventions. Conservative management of symptomatic LHB brachii tendinopathy is usually considered the first-line treatment, such as rest, non-steroidal anti-inflammatory drugs (NSAIDs), corticosteroid injection, and physical therapy [2, 6, 7]. NSAIDs are the most commonly prescribed drugs for treatment of tenosynovitis [8], which can significantly reduce the intensity of pain and improve the range of motion. However, the therapeutic effect will be suppressed by poor tolerance, obvious gastrointestinal reactions, hepatorenal toxicity, and adverse events with cardiovascular risk [9–11]. Failure of conservative treatments may warrant surgical interventions. Surgical treatments include long head biceps tenotomy and tenodesis, which are the most common surgical procedures on the treatment of tenosynovitis in clinic [7]. However, studies have reported that surgery also has many limitations, including pain recurrence after operation and varying degrees of joint deformities [6, 12, 13]. In addition, due to patient’s fear of surgery, many patients with tenosynovitis of LHB brachii will not select surgery as their first treatment choice.

Apart from the abovementioned methods of treatment for tenosynovitis of LHB brachii, complementary and alternative therapies, such as acupuncture and moxibustion, tuina, and Chinese herbs, have been used to alleviate pain and improve the function of LHB brachii. As one of the most important methods in traditional Chinese medicine, acupuncture has been used to treat anterior pain of shoulder in China for a long history, and some researchers pointed out that acupuncture has promising efficacy in the treatment of tenosynovitis of LHB brachii and costs less compared to other treatment methods [14–18]. In further analyses, although different studies have shown different effects of acupuncture on the treatment of tenosynovitis of LHB brachii [17, 19], the effectiveness and safety of acupuncture for treating tenosynovitis of LHB brachii remain uncertain and current evidence is limited. Moreover, to date, there are no systematic reviews to evaluate the efficacy of acupuncture in the treatment of tenosynovitis of LHB brachii, so a critical examination of the evidence regarding acupuncture for treating tenosynovitis of LHB brachii is warranted.

In our study, we will perform the first systematic review and meta-analysis to investigate the efficacy and safety of acupuncture on the treatment of tenosynovitis of LHB brachii. Furthermore, we expect that the results will provide evidence to clarify the current controversy surrounding acupuncture on the treatment of tenosynovitis of LHB brachii. In addition, based on our summary of the literature, we hope it can guide clinical practice of acupuncture on the treatment of tenosynovitis of LHB brachii in the future.

## Methods

### Study registration

The protocol of this review will be conducted and reported in accordance with the Preferred Reporting Items for Systematic Reviews and Meta-Analysis Protocols (PRISMA-P) statement guidelines [20]. The protocol has been registered on the International Prospective Register of Systematic Reviews (PROSPERO), and the trial registration number is CRD42020167434.

### Inclusion criteria for study selection

#### Type of studies

We will estimate the publications according to the criteria of the review objectives and participants, interventions, comparisons, outcomes (PICO). Only randomized controlled trials (RCTs) involving acupuncture against with other treatments, placebo treatment, or sham treatment in patients with tenosynovitis of LHB brachii will be included in this review. Language of literature will be limited to Chinese and English. Studies that mentioned the term of “randomization” will also be considered, while studies using incorrect randomization methods will be excluded. Additionally, other designs such as in vivo, in vitro, case report, and non-RCTs will also be excluded.

#### Type of participants

Studies that enrolled patients with tenosynovitis of LHB brachii, regardless of gender, age, race, or nationality, who received acupuncture therapy (with or without other treatment) will be included. However, the patients with tendon fracture will not be taken into account. Besides, patients with other serious illnesses, such as bone tuberculosis, cancer, cardiovascular disease, and liver and kidney disease, will be excluded.

#### Type of interventions

We will consider studies evaluating the following treatments including body acupuncture (MA or EA), auricular acupuncture, scalp acupuncture, and warm needle acupuncture. In addition, studies involving acupuncture as the sole treatment or combined with other therapies that were equally used in both experiment and control group will be included in this review.

#### Type of comparators or control

We will include and classify the comparators in study as follows: (1) acupuncture versus invasive sham/minimal acupuncture, (2) acupuncture versus non-invasive placebo acupuncture, and (3) acupuncture versus waiting list/usual care/no treatment. Articles comparing different acupoints or different forms of acupuncture will be excluded.

All inclusion and exclusion criteria for considering studies for this review are summarized in Supplementary Table 1.

### Outcome measures

#### Primary outcomes

The visual analog scale (VAS), the Melle score of shoulder joint functional activity, and the ability assessment of daily living activities (ADL) will be regarded as the primary outcomes.

#### Secondary outcomes

The secondary outcomes will be assessed by shoulder range of motion, including flexion, extension, abduction, adduction, external rotation, and internal rotation. In addition, secondary outcomes include quality of life that was assessed by well-acknowledged scales, such as the 36 Short-Form Health Survey (SF-36). Furthermore, adverse events caused by acupuncture or other therapies in the studies will also be recorded.

### Search strategies

An electronic search will be carried out in the following databases, including PubMed, Web of science, EMBASE, the Cochrane Library, Chinese National Knowledge Infrastructure (CNKI), Chinese Biomedical Literature Database (CBM), WanFang Database, and Technology Periodical Database (VIP). Terms of medical subjects (MeSH) and keywords will be used individually or in combination during the query. The specific search strategy for PubMed will be taken as an example, which will be shown in Table 1. Nevertheless, the searching strategy for other databases will be minorly modified. In addition, Chinese characters with the same meaning will be used for literature retrieval in the Chinese databases.

**Table 1:** Search strategy used in PubMed database

No.	Search items
#1	Randomized controlled trial [pt]
#2	Controlled clinical trial [pt]
#3	Randomized [tiab]
#4	Placebo [tiab]
#5	Clinical trials [MeSH]
#6	Randomly [tiab]
#7	Trial [ti]
#8	#1 OR #2 OR #3 OR #4 OR #5 OR #6 OR #7
#9	Humans [MeSH]
#10	#8 AND #9
#11	Tenosynovitis of long head of biceps or tenosynovitis of LHB [MeSH]
#12	Tenosynovitis of biceps or tenosynovitis [ti, ab]
#13	Tendinitis of long head of biceps or tendinitis of LHB [MeSH]
#14	Tendinitis of biceps or tendinitis [ti, ab]
#15	#11 OR #12 OR #13 OR #14
#16	Acupuncture therapy OR electroacupuncture therapy [MeSH]
#17	(Acupuncture OR electroacupuncture OR electro-acupuncture OR manual acupuncture OR auricular acupuncture OR warm needling) [ti, ab]
#18	#16 OR #17
#19	#10 AND #15 AND #18

Moreover, we will filter relevant medical journals and magazines to clarify literature which is not included in the electronic databases. Meanwhile, clinical trial registries, like the WHO International Clinical Trial Registry Platform, Chinese Clinical Registry, and ClinicalTrials.gov, will be searched for ongoing trials with unpublished data. The reference lists of all potential publications, including relevant systematic reviews, will be manually retrieved and reviewed to further locate additional trials. Incomplete data will be obtained via contacting the corresponding authors.

### Study collection

**Fig. 1:**
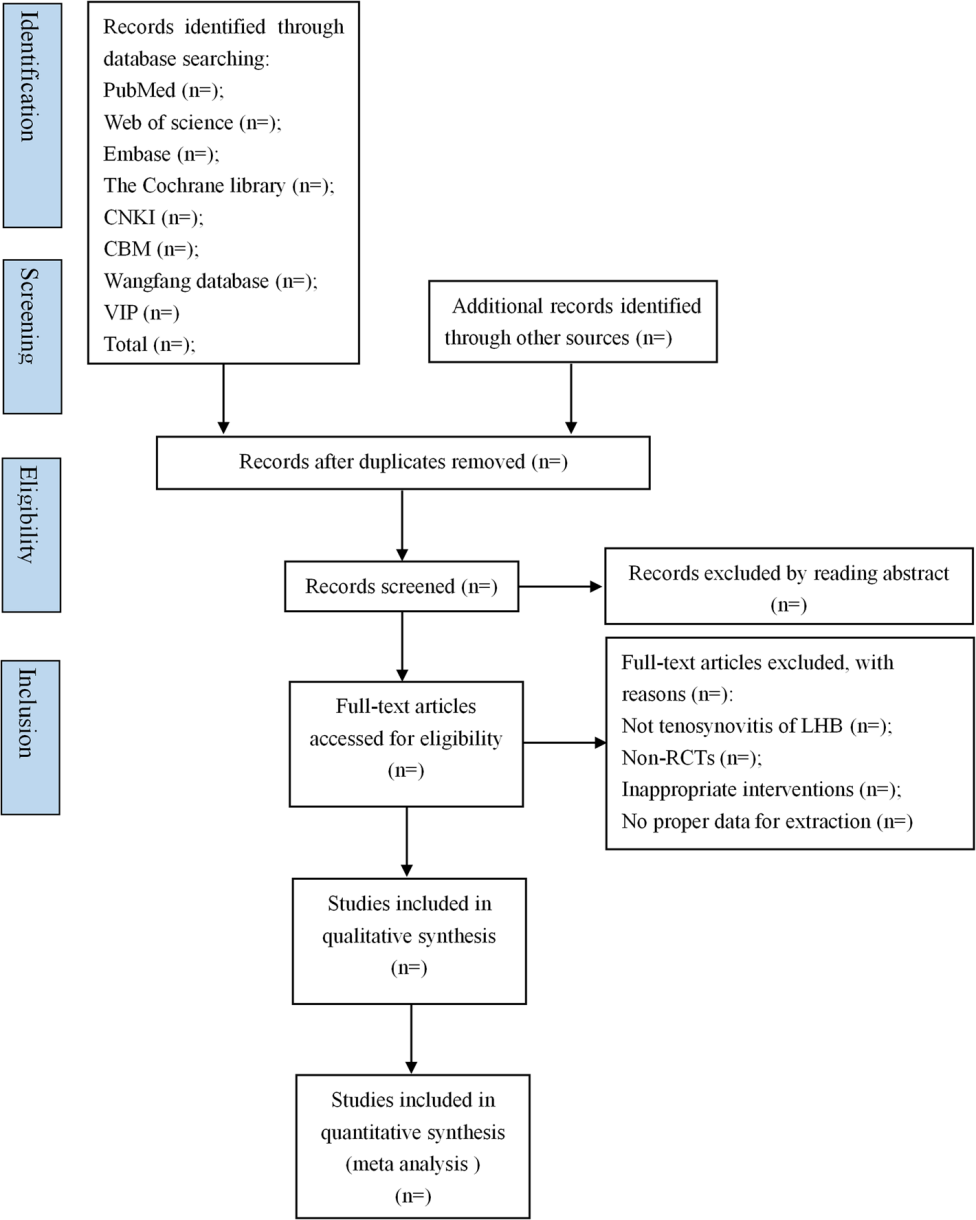
Flow diagram of the study selection process

Two reviewers (RRL and YLJ) will evaluate the title and abstract of all studies for possible candidates, respectively. Any duplicate studies will be removed. After title and abstract screening, the full-text copies of all eligible studies will be downloaded for re-evaluation. Once the reviewer is uncertain about the eligibility of any study, its full text will be obtained to re-examine. An additional reviewer (XFH or RJH) will be consulted in case of disagreement. Excluded studies and the reasons of exclusion will be recorded. The specific process of study screening will be displayed in a Preferred Reporting Items for Systematic Reviews and Meta-Analyses (PRISMA). The flow diagram of all study selection procedure is shown in Fig. 1.

### Data extraction

Two investigators (RRL and RJH) will independently perform data extraction. The general information, participants, methods, interventions, outcomes, results, adverse events, conflict of interest, ethical approval, and other information will be extracted. Furthermore, we will contact the authors for further information when the reported data is not sufficient. Besides, any disagreement will be resolved by discussion between the two authors (RRL and RJH) and further disagreements will be arbitrated by the third author (JQF).

### Dealing with missing data

The corresponding authors or relevant authors will be contacted by telephone or e-mail for insufficient or missing data. In case of no reply from the authors or missing data cannot be supplied, the missing data with replacement values will be imputed, treating these as if they were observed. The last observation carried forward imputation method will be used to assume a missing value, and then, an intention-to-treat analysis will be performed. Moreover, if possible, the sensitivity analyses will be performed to assess how sensitive the results are to reasonable changes in the assumptions that are made. The potential impact of missing data on the final outcome of the review will be addressed in the discussion.

### Risk of bias assessment

Two authors (RRL and YLJ) will assess the risk of bias with the Cochrane Collaboration’s tool for risk of bias assessment in all included studies [21]. The following domains for risk of bias will be evaluated: sequence generation, allocation sequence concealment, blinding of participants and personnel and outcome assessors, incomplete outcome data, selective outcome reporting, and other sources of bias. The judgment on these items will be classified into three levels: “low risk of bias,” “high risk of bias,” or “unclear risk of bias.” The conflicts or any discrepancies will be resolved by discussion or will be judged by another reviewer (JQF) to achieve the consensus.

### Quality of evidence assessment

According to Grading of Recommendations Assessment, Development, and Evaluation (GRADE) method [22], the evidence quality evaluation of key outcomes can be regarded as four levels: high quality, moderate quality, low quality, and very low quality [23]. Evidence quality is generally judged on the basis of risk of bias, inconsistency, indirectness, inaccuracy, and publication bias.

### Measures of treatment effects

We will use the Review Manager software 5.3 (V.5.3) [24] to carry out statistical analysis. Mean difference (MD) or standardized mean difference (SMD) will be used for continuous data. In addition, risk ratio (RR) or risk difference (RD) will be used for the analysis of dichotomous data. Furthermore, the corresponding 95% confidence interval (CI) for each parameter will be calculated between the treatment group and the control group.

### Assessment of heterogeneity

The heterogeneity will be assessed by *I*^2^ statistical test. If the *I*^2^ test is less than 50%, the fixed-effect model will be used for data synthesis. Otherwise, the random-effects model will be conducted with heterogeneous data, which *I*^2^ test is between 50 and 75%. If the *I*^2^ test is higher than 75%, we will find the possible reasons from both clinical and methodological perspectives and provide an explanation or conduct subgroup analysis.

### Assessment of reporting bias

A funnel plot will be generated to assess reporting bias when more than 10 trials are included [25].

### Data synthesis

Clinical data will be imported into the RevMan software (V.5.3) to perform data synthesis, and the significance threshold will be *P* < 0.05 on two sides. A forest plot for each parameter will be constructed to indicate the weight ratio of each incorporated study.

### Sensitivity analysis

Sensitivity analysis will be conducted to monitor the robustness of primary decision made in the review process. Several decision nodes, such as sample size, methodological weakness, and missing data, will be considered. The results of the sensitivity analysis will be presented in summary tables. The risk of bias in the review process as indicated by the results of the sensitivity analysis will be discussed.

### Subgroup analysis

If data is available, a subgroup analysis will be conducted according to variations in the characteristics of the trial participants and acupuncture treatment. When considerable heterogeneity is detected in a previous analysis, a subgroup analysis will be performed if necessary.

### Ethics and dissemination

Ethics approval will not be necessary, because the included publications in our study do not involve patients’ individual privacy. The main data will be extracted from published literature.

This systematic review will be published in a peer-reviewed journal or conference report to provide a reference for the effectiveness and safety of acupuncture treatment for tenosynovitis of LHB brachii.

## Discussion

The proximal tendinous portion of LHB brachii muscle is a common cause of anterior shoulder pain [26]. However, it is unclear whether the pain manifestation was associated with acute inflammation or with chronic degenerative changes of LHB tendon [27]. The tenosynovitis of LHB brachii always seriously leads to the result of pain; especially, it could be aggravated by motion of internal rotation and back extension. Hence, the range of motion of the shoulder joint was limited and subsequently affected the quality of people’s daily life [2]. Conventional treatment of western medicine and surgical intervention on tenosynovitis of LHB brachii significantly relieved the symptoms and improved the people’s life quality [28–30]. However, adverse events and side effects caused by the abovementioned treatments may affect the therapeutic effects. Therefore, it is necessary to seek alternative therapy with higher effective rate and less adverse effects.

Well-established acupuncture therapy is a reliable method to relieve pain; it could not only treat the tenosynovitis of LHB brachii caused by serious sports injury, but also have a good effect on the anterior shoulder pain caused by degeneration. Unfortunately, there is no comprehensive systematic review to provide enough evidence for acupuncture treatment for tenosynovitis of LHB brachii. Hence, carrying out a systematic review and meta-analysis from available literature to evaluate the effectiveness and safety of the acupuncture treatment on tenosynovitis of LHB brachii is warranted. The protocol of this systematic review and meta-analysis will include and integrate the latest and most comprehensive literature in this field, hoping to provide an objective treatment method for patients with tenosynovitis of LHB brachii and inspire more peer experts to implement more relevant clinical trials in the future.

However, there are some limitations in this study. Firstly, there are not enough publications on the treatment of LHB brachii tenosynovitis by using acupuncture. Hence, further correlated analysis, such as subgroups analysis, sensitivity analysis, and test for publication bias, could not be carried out because of the insufficient numbers of included trials. Secondly, we will only acquire the studies that were reported in Chinese or English; databases in other language such as Japanese or Korean will not be involved, which may lead to language bias.

In summary, this is the first systematic review and meta-analysis to ascertain the effectiveness and safety of the acupuncture treatment on tenosynovitis of LHB brachii. Based on rigorous study design and accurate evaluation of the literature, we expect that the review will provide evidence of acupuncture treatment on tenosynovitis of LHB brachii.

## Supplementary information

**Additional file 1: Supplementary Table 1.** Inclusion and exclusion criterion for considering studies for this review.

## Data Availability

This protocol also has been registered on the International Prospective Register of Systematic Reviews, and the trial registration number is CRD42020167434 (https://www.crd.york.ac.uk/PROSPERO/#myprospero).
