# Supplementary material for: Effectiveness and safety of tenosynovitis of the long head of the biceps brachii with acupuncture: a protocol for a systematic review and meta-analysis
Source: Trials. 2020 Oct 20;21:869. doi: 10.1186/s13063-020-04800-6 (PMC7576742; doi:10.1186/s13063-020-04800-6)
Supplement: Supplementary file 1 — Additional file 1: Supplementary Table 1. Inclusion and exclusion criterion for considering studies for this review. [file 13063_2020_4800_MOESM1_ESM.doc]

**Supplementary Table 1.** **Inclusion and exclusion criterion for considering studies for this review**

| **Study selection** | **Inclusion** | **Exclusion** |
| --- | --- | --- |
| Studies | 1. RCTs involving acupuncture against another treatment or placebo/sham in patients with tenosynovitis of LHB brachii. 2. Studies that the term of “randomization”was mentioned. 3. Studies that were reported in Chinese or English. | 1. Incorrect randomization methods. 2. Other designs (such as in vivo, in vitro, case report and non-RCTs) |
| Participants | Patients with tenosynovitis of LHB brachii who received acupuncture therapy. | 1. Tendon fracture. 2. Other serious illnesses (such as bone tuberculosis, cancer, cardiovascular disease, liver and kidney disease) |
| Interventions | 1. Body acupuncture, including MA or EA. 2. Auricular acupuncture. 3. Scalp acupuncture. 4. Warm needle acupuncture. 5. Acupuncture combined with other therapies. | Other therapies |
| Comparators or control | 1. Invasive sham/minimal acupuncture 2. Non-invasive placebo acupuncture 3. Waiting list 4. Usual care 5. No treatment | 1. Comparing different acupoints 2. Different forms of acupuncture |

**(Abbreviations: RCTs = Randomized Controlled Trials, LHB = Long Head of Biceps,**

**MA = Manual Acupuncture, EA = Electro-acupuncture)**
